# Red LED Light Improves Pepper (*Capsicum annuum* L.) Seed Radicle Emergence and Growth through the Modulation of Aquaporins, Hormone Homeostasis, and Metabolite Remobilization

**DOI:** 10.3390/ijms24054779

**Published:** 2023-03-01

**Authors:** Chokri Zaghdoud, Irene Ollio, Cristóbal J. Solano, Jesús Ochoa, Juan Suardiaz, Juan A. Fernández, María del Carmen Martínez Ballesta

**Affiliations:** 1 Bureau de Transfert de Technologie (BuTT), Université de Gafsa, Gafsa 2112, Tunisia; 2 Ingeniería Agronómica, Technical University of Cartagena, Paseo Alfonso XIII 48, E-30203 Cartagena, Spain; 3 Recursos Fitogenéticos, Instituto de Biotecnología Vegetal, Edificio I+D+i, E-30202 Cartagena, Spain; 4 División of Innovation in Telematic Systems and Electronic Technology (DINTEL), Technical University of Cartagena, Campus Muralla del Mar, s/n, E-30202 Cartagena, Spain

**Keywords:** aquaporins, *Capsicum annuum*, LED lighting, radicle emergence, seed germination, water uptake

## Abstract

Red LED light (R LED) is an efficient tool to improve seed germination and plant growth under controlled environments since it is more readily absorbed by photoreceptors’ phytochromes compared to other wavelengths of the spectrum. In this work, the effect of R LED on the radicle emergence and growth (Phase III of germination) of pepper seeds was evaluated. Thus, the impact of R LED on water transport through different intrinsic membrane proteins, via aquaporin (AQP) isoforms, was determined. In addition, the remobilization of distinct metabolites such as amino acids, sugars, organic acids, and hormones was analysed. R LED induced a higher germination speed index, regulated by an increased water uptake. *PIP2;3* and *PIP2;5* aquaporin isoforms were highly expressed and could contribute to a faster and more effective hydration of embryo tissues, leading to a reduction of the germination time. By contrast, *TIP1;7*, *TIP1;8*, *TIP3;1* and *TIP3;2* gene expressions were reduced in R LED-treated seeds, pointing to a lower need for protein remobilization. *NIP4;5* and *XIP1;1* were also involved in radicle growth but their role needs to be elucidated. In addition, R LED induced changes in amino acids and organic acids as well as sugars. Therefore, an advanced metabolome oriented to a higher energetic metabolism was observed, conditioning better seed germination performance together with a rapid water flux.

## 1. Introduction

Seed germination is usually considered the most critical stage in seedling establishment, which determines successful crop yield and quality [1]. The process of germination begins with the seed imbibition of water, triggering arrested physical and metabolic activities, including cell expansion, cell division, and reserve mobilization [2], and ends with radicle protrusion [3]. Generally, seeds uptake water during germination in a typical triphasic pattern, initiating with a rapid water absorption (Phase I, imbibition) followed by a plateau phase (Phase II, little net water uptake with metabolic preparation for radicle protrusion), and a second burst of water uptake (Phase III, coupled with radicle emergence) [4]. The micropyle and hilum of seeds are the main regions involved in the absorption of water [5], which is moved into the seed tissue and organs via three different pathways: apoplastic, symplastic, and transcellular paths. The movement of water and other small neutral molecules via the transcellular path—traversing through cell membranes—involves aquaporins (AQPs), which are water-selective channels [6,7]. In higher plants, AQPs are divided into five major subfamilies: plasma membrane intrinsic proteins (PIPs), tonoplast intrinsic proteins (TIPs), NOD26-like intrinsic proteins (NIPs), small basic intrinsic proteins (SIPs), and the uncategorized X intrinsic proteins (XIPs) [8]. In turn, these subfamilies can be further divided into multiple isoforms, depending on the localization and functional properties [9]. Numerous studies have showed the important function of orthodox seed AQPs in water imbibition and subsequent germination, with the expression patterns being spatiotemporal specific at the transcription level [10,11,12]. Indeed, the dynamics of gene expression during germination reveals the implication of two time-separated sets of AQPs [13]: (i) the first set of AQPs is potentially involved in the initial germination process, being the transcript level abundant in dry seed just before initial rehydration, and dramatically reduced during imbibition and germination; and (ii) the second set of AQPs drives the rapid cellular expansion and embryo growth, where the transcripts are increased at, or immediately after, germination. The low levels of AQP gene expression during Phase I of germination [12,14,15] are attributed to the dry seed AQP transcripts stored, ready for immediate translation upon initial rehydration [13,16,17], rather than AQPs non-functionality. PIPs and TIPs are the predominant AQPs in plant seeds. However, whereas PIPs function as water uptake and transport across cell-plasma membranes during seed imbibition and the early growth of the embryo [18], seed-specific TIPs act mainly from the beginning of Phase II as markers of protein remobilization to maintain turgor pressure [12,19]. During Phase II, vacuolation occurs through transition from cells small protein storage vacuoles (PSVs) to large central lytic vacuoles (LVs), leading to significant increase in cellular turgor pressure and the activation of several hydrolytic enzymes [11,20]. This transition was accompanied with a shift from *TIP3s* to *TIP1*/*TIP2* gene expression [17,19]. TIP3 are enriched in PSVs, whereas TIP1 and TIP2 are preferentially localized in LVs and vegetative storage protein vacuoles, respectively [21], which revealed implication of these isoforms in protein mobilization, a process that is crucial for embryo-cell elongation and radicle emergence [22]. Nevertheless, the dual localization of TIP3 in tonoplast and plasma membrane highlights their role in favouring optimal water uptake during seed imbibition [20]. In addition, transcriptionally, *TIP4;1* expression begins post germination [23].

Amino acids play vital roles in the metabolism of seeds during germination. They are used for the synthesis of seed-storage proteins and are precursors of secondary metabolites that are a source of energy for seeds [24]. Also, organic acids may represent the store pools of carbon in metabolic pathways, taking part in the mobilization of stored lipids and carbohydrates during seed germination [25].

Finally, seed germination is also controlled by a crosstalk between plant hormones, including gibberellins (GAs), abscisic acid (ABA), ethylene (ET), auxins (AUX), cytokinins (CKs), and brassinosteroids (BRs) [26], and metabolizable sugars derived from storage that appear to have both nutritional and signalling stimulant functions [27]. ABA specifically inhibits the endosperm rupture and Phase III water uptake by the emerging embryo, but does not alter the spatial and temporal pattern of Phase I and II water uptake [28]. In contrast, GAs, ethylene, and BRs enhance germination by antagonistically suppressing ABA, weakening the tissue surrounding the embryo, which increases the embryonic growth potential, thereby helping radicle protrusion [29,30]; the signal-transduction pathways and mechanisms are hormone specific [31]. GAs and ABA antagonistically modulated α-amylase activity and the transformation of small PSVs into LVs in the aleurone cells of seed endosperm, with GA and ABA acting as an inducer and inhibitor of this transformation, respectively [32]. AUXs (particularly indole-3-acetic acid [IAA]) are involved in the cell-wall loosening and polysaccharide mobilisation mediated by hydroxyl radicals (^•^OH), processes needed for cell elongation and division [33]. Interestingly, Finkelstein and Lynch [27] reported that glucose suppressed the inhibitory effects of ABA on radicle emergence in a light-dependent manner, although the genetic components involved were not identified. During Phase III of germination, sucrose and hexose transporters, as well as H^+^-ATPase, become the fundamental proteins involved in the active cell elongation to support radicle extension [34]. Hormone-driven regulatory responses also control water uptake during seed germination through the regulation of AQP gene expression [13].

In small-seeded plants such as Arabidopsis, lettuce, tomato [35,36], and pepper [37], germination was under phytochrome-mediated photocontrol, with light being a critical environmental determinant during this process. Recently, the use of light-emitting diodes (LEDs) has spread to plant production purposes; they are more efficient than fluorescent lamps [38], since they allow a tight control of waveband emission and light intensity with low energy consumption [39]. Red (R, 600–700 nm) and blue (B, 400–500 nm) lights are the most-used source of radiation for plant production in a controlled environment, as these wavelengths are most readily absorbed by photoreceptor phytochromes [40]. Thus, R and B LED lights were more efficient for seed germination and plant growth than other wavelengths, such as green (500–600 nm) and far red (700–800 nm) [41]. The effects of LED light on seed germination are species specific and spectra dependent [42]. Indeed, when lettuce (*Lactuca sativa* L.) seeds were exposed to different LED light wavelengths, the highest induction of germination was observed in cv. Banchu Red Fire under R light (at 50 Hz and duty ratio of 20%) [43], and in cv. Levistro under lights with a higher B component [44]. In oilseed rape (*Brassica napus* ‘Modena’), R (638 nm) and B (450 nm) LED lights significantly increased and decreased the seed germination percentage, respectively [45]; whereas, at the same intensity and photoperiod, both LED lights inhibited germination in soybean (*Glycine max*) seeds [46]. Cho et al. [47] reported that light enhanced the germination rate through the activation of photoreceptor phytochrome B, which mediates GA signalling and metabolism. Most reports on the effect of LED lights on germination have studied seed parameters (germination percentage, GP; germination speed index, GSI; radicle length, RL; hypocotyl length, HL). However, to the best of our knowledge, no works were done illustrating their impact on water uptake and radicle emergence processes with the involvement of aquaporins.

Since water plays a crucial role in germination, the aim of this study was to investigate the relationship between better germination performances, through the application of Red LED irradiation, and seed water status. In addition, AQP isoforms involved in water transport will be determined, as well as the changes in the hormone and metabolite profiles under these conditions.

## 2. Results

### 2.1. R LED Light Accelerates Seed Hydration and Suppresses the Inhibitory Effect of Azide during Germination

**Figure 1:**
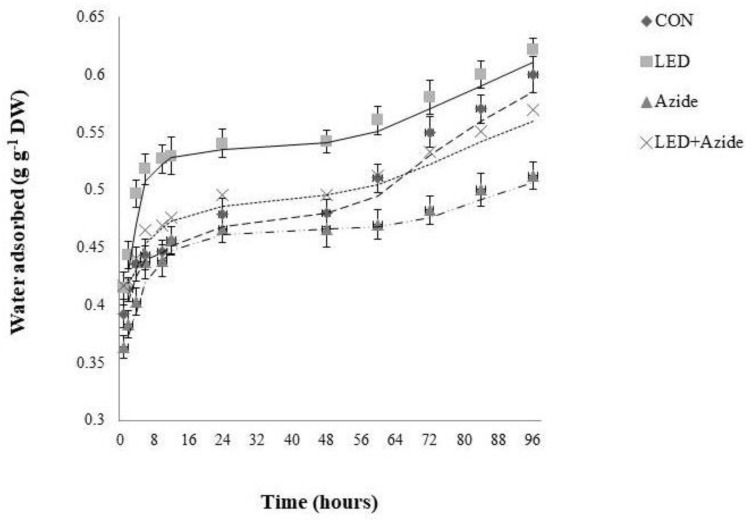
The water adsorption kinetics (g g^−1^ DW) of pepper seeds during the imbibition initial phase (Phase I), plateau phase of germination (Phase II), and radicle emergence and growth (Phase III) under Control (CON, unlighted seeds without azide), previous pulses of R LED irradiation (660 nm) (LED, without azide), azide (azide, unlighted seeds), and azide R LED-irradiated (LED+azide) conditions. Data are the means of five repetitions (*n* = 5).

The kinetics of water uptake by pepper seeds during Phases I, II, and III of germination (from 0 to 96 h) were monitored in the presence or absence of azide, with or without a previous pulse of R LED irradiation (Figure 1). Upon exposure to sufficient moisture, the water content for the different treated seeds showed a rapid increase during the first 6 h of imbibition, followed by a gradual progression from 6 h to 10 h, reaching a plateau phase (Phase II), then the curve of variation stabilized from 10 h to 48 h. After radicle emergence (at 48 h), a second gradual increase (Phase III) was reached until 96 h, when radicles were 1 cm long.

In all phases, hydration was faster in R LED-irradiated seeds in comparison with the rest of the treatments. Seed hydration was the lowest under azide treatment (7.35% lower than Controls after the first 6 h). However, a pulse of R LED irradiation before azide addition suppressed the inhibitory effect of azide on seed imbibition, with the values of water uptake under combined R LED and azide treatments similar to those of Controls.

### 2.2. R LED Light Increases the Radicle Emergence Rate, Germination Percentage, Germination Speed Index (GSI), and Humidity Percentage

**Figure 2:**
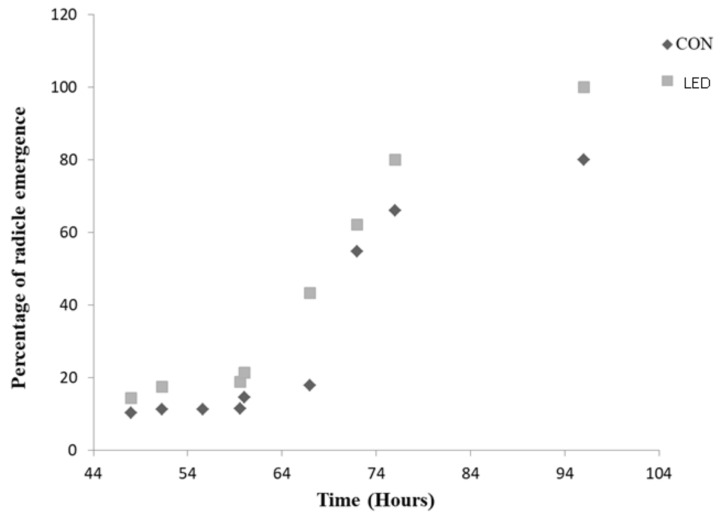
Percentage of radicle emergence (from 48 h to 96 h) in Control (CON, unlighted) and irradiated R LED (LED) pepper seeds after Phases I and II of germination.

A time course (from 48 h to 96 h) of the percentage of radicle protrusion was obtained for Control and R LED-irradiated seeds (Figure 2). At all measured time points, the highest rates of radicle emergence were recorded for seeds previously irradiated by R LED light. For both treatments, the rates of radicle emergence followed a sigmoid pattern of evolution, with an exponential behaviour starting from 60 h for R LED-treated seeds and from 67 h for Control. At 67 h of imbibition, the percentage of emerged radicles was 2.42-fold higher in R LED-lighted seeds, compared to Control.

The tetrazolium test showed no statistical differences regarding the percentage of embryo viability between Control and R LED-irradiated seeds (Table 1), reflecting the innocuous effect of R LED light on seed tissues. R LED-irradiated seeds showed at 96 h a significantly increased germination percentage (GP) and higher germination speed index (GSI) than Controls, by about 5.7%, that together with a significantly higher percentage (~23.86%) of humidity after imbibition during 96 h, and subsequent lyophilisation, reflect the higher ability of R LED-treated seeds to adsorb water faster.

### 2.3. Seeds AQP Isoforms Were Differentially Expressed under Control and R LED-Irradiation during Phase III

**Figure 3:**
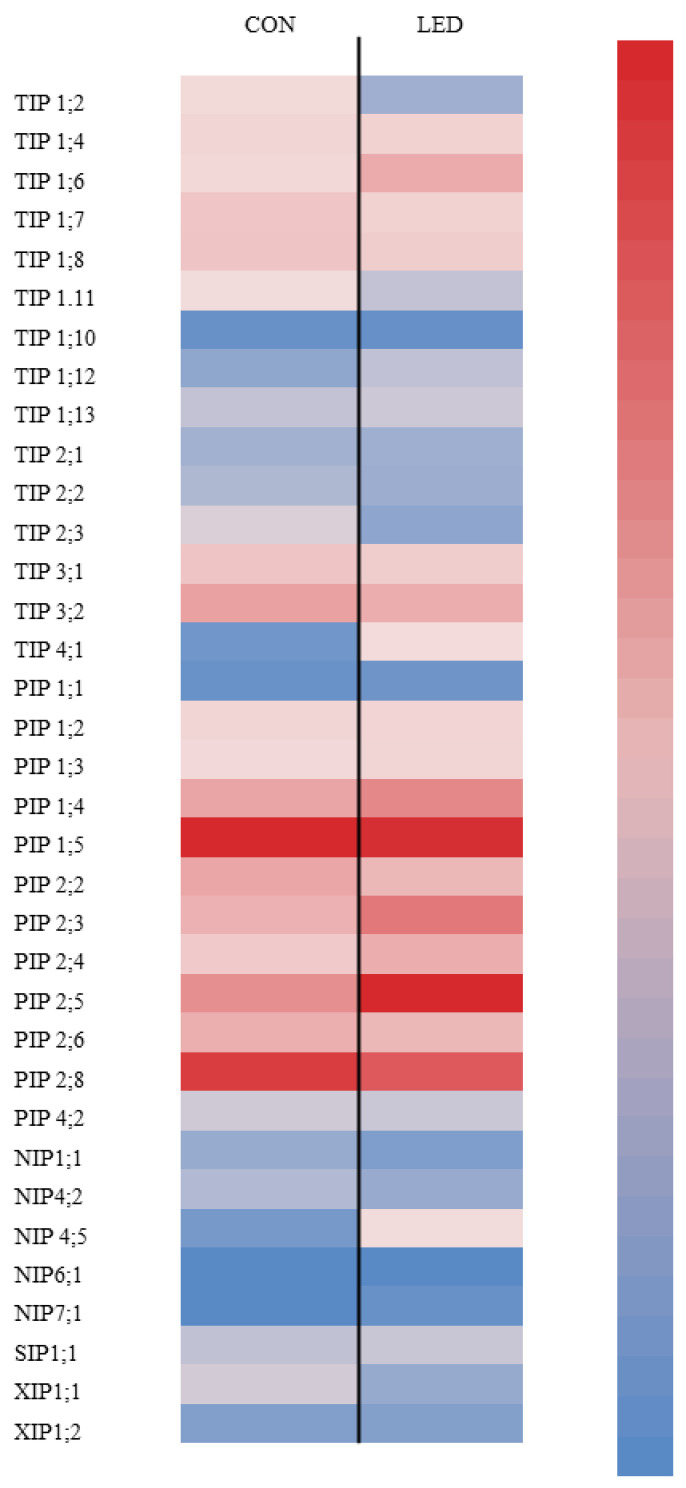
Heat-map representation of *TIP*, *PIP*, *NIP*, *SIP*, and *XIP* isoforms expression in Control (CON, unlighted) and irradiated R LED (LED) pepper seeds, after radicle emergence during germination (at 96 h of imbibition), as compared to the *NIP1;1* (lower expression) of each treatment. Data are means of three repetitions (*n* = 3).

The expression of different AQP isoforms was determined in seeds with protruding radicles after 96 h of imbibition in water. *NIP1;2*, *NIP3;1*, *NIP4;1*, *NIP4;6*, and *TIP5;1* isoforms were not detected in our sweet pepper seeds. A heat-map graph characterizes the relative expression profile of detected isoforms: 12 *PIPs*, 15 *TIPs*, 5 *NIPs*, 2 *XIPs*, and 1 *SIP* in Control and R LED-irradiated pepper seeds (Figure 3). This map reflects the isoforms’ expression relative to *NIP1;1* expression of each individual treatment. Transcript analysis demonstrated that, for both treatments, most *PIP* and *TIP* subfamily genes were much more highly expressed than *NIP*, *SIP*, and *XIP* subfamily genes, being the expression treatment specific. In Control seeds, *TIP1;7*, *TIP1;8*, *TIP3;1*, and *TIP3;2* were the *TIP* isoforms that showed the highest expression levels; among *PIPs*, *PIP1;4*, *PIP1;5*, *PIP2;3*, *PIP2;5*, and *PIP2;8* were the highest expressed. In R LED-lighted seeds, in addition to those mentioned for Controls, other AQPs isoforms showed high expression compare to *NIP1;1* including *TIP1;4*, *TIP1;6*, *TIP4;1*, and *PIP2;3*.

**Figure 4:**
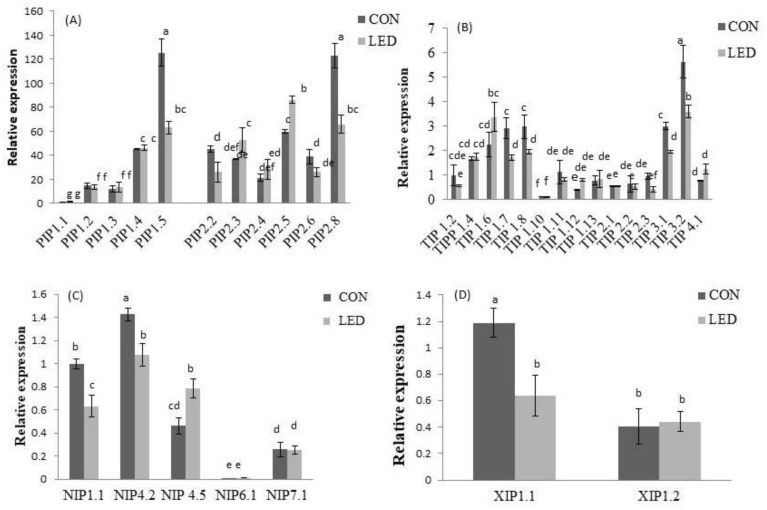
Relative expression of (**A**) *PIP*, (**B**) *TIP*, (**C**) *NIP*, and (**D**) *XIP* isoforms for Control (CON, unlighted) pepper seeds and irradiated R LED (LED) pepper seeds, after radicle emergence during germination (at 96 h of imbibition). Bars are means ± SE (*n* = 3). The different letters indicate significant differences (*p* < 0.05, Tukey’s test) between treatments.

The expression profiling of AQP isoforms, comparing both Control and R LED light treatments, were also determined at 96 h of pepper seed imbibition (Figure 4). Comparison between isoforms was realized considering *NIP1;1* expression of CON rootlets as reference for both treatments. Regarding *PIP* gene expression (Figure 4A), significant differences among the treatments were found for *PIP2;3* and *PIP2;5*, which showed a significant increase in expression (by 43.99% and 44.02%, respectively) in irradiated R LED seeds with respect to the Control, and *PIP1;5* and *PIP2;8*, which had a significant decrease in expression by 49.71% and 41.73%, respectively. Among the *TIP* isoforms, significant differences between treatments were observed for *TIP1;7*, *TIP1;8*, *TIP3;1*, and *TIP3;2* gene expressions, where reductions by about 41.08%, 34.43%, 34.99% and 37.24%, respectively, were recorded in R LED-lighted seeds, compared to Controls (Figure 4B). The expression profiling of *NIPs* showed that *NIP1;1* and *NIP4;2* isoforms were also significantly lower expressed (by 35.12% and 24.75%, respectively) in seeds previously exposed to R LED irradiance, relative to Controls, while *NIP4;5* was significantly higher expressed by 70.91% (Figure 4C); *NIP6;1* and *NIP7;1* expression did not differ significantly among the treatments. Significant differences in *XIP* expression were only observed for *XIP1;1*, which showed an increase in expression by about 46.72% in irradiated seeds with respect to the Controls (Figure 4D). *SIP1;1* was detected but no significant differences were found between both treatments.

### 2.4. R LED Irradiance Modulates the Levels of Phytohormones during Phase III

Significant differences (*p* < 0.05) in the hormone levels in pepper seeds with protruding radicles—after 96 h of imbibition—among Control and R LED-lighting treatments were observed with changes in the amount of salicylic acid (SA), indole-3-butyric acid (IBA), ABA, and jasmonic acid (JA) (Table 2). Endogenous levels of SA were significantly increased (by about 32.03%) in R LED-irradiated seeds compared to Controls, while those of JA were significantly decreased (by 11.65%). Interestingly, IBA appeared only in R LED-irradiated seeds and ABA was found exclusively in Control seeds. IAA, GA3, and 6-BA were not detected (n.d).

### 2.5. R LED Light Induces Changes in the Metabolite Content during Phase III

#### 2.5.1. Sugars

**Figure 5:**
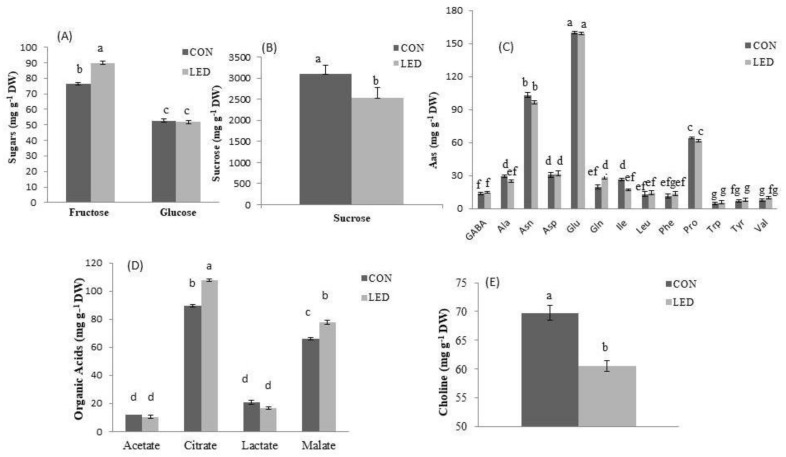
Concentrations (mg g^−1^ DW) of (**A**) sugars, (**B**) sucrose, (**C**) amino acids (Aas), (**D**) organic acids, and (**E**) choline in Control (CON, unlighted) pepper seeds and in seeds previously irradiated with a pulse of R LED light (LED) after radicle emergence during germination (at 96 h of imbibition). Bars are means ± SE (*n* = 5). Different letters denote a statistically significant difference (*p* < 0.05, Tukey’s test) between treatments.

The sugars quantified with 1H-NMR in the seeds of sweet pepper were fructose, glucose, and sucrose (Figure 5A,B). Myo-inositol was not detected for any treatment in the pepper seeds, although its determination was also carried out. Of those quantified, the highest amount quantified was for sucrose (Figure 5B), followed by fructose and glucose (Figure 5A).

For all of them, except glucose, the R LED irradiation affected their concentration. Regarding to Control, previously R LED irradiated pepper seeds had a significant percentage of 8.53% lower sucrose content after radicle emergence during germination (at 96 h of imbibition) (Figure 5B), and 17.55% significantly higher fructose concentration (Figure 5A). However, no statistical differences were observed between treatments with regard to glucose levels.

#### 2.5.2. Amino Acids

The amino acids detected with 1H-NMR in pepper seeds were GABA, Alanine (Ala), Asparagine (Asn), Aspartate (Asp), Glutamate (Glu), Glutamine (Gln), Isoleucine (Ile), Leucine (Leu), Phenylalanine (Phe), Proline (Pro), Tryptophan (Trp), Tyrosine (Tyr), and Valine (Val). The most abundant transported amino acids with a high N:C ratio during germination in seeds were Glu followed by Asn and Pro (Figure 5C).

In general, no changes in the amino acids content were found in R LED-irradiated seeds compared to Control seeds. However, significant differences between treatments were recorded for Ala and Gln, which showed significantly lower concentrations (reductions of 10.42% and 38.93%, respectively) in R LED-lighted seeds, compared to Controls, and Ile, of which the concentration was significantly lower by about 9.52% (Figure 5C). In addition, the other major forms of amino acids, Asn and Pro, showed similar seed levels under both treatments (Figure 5C).

#### 2.5.3. Organic Acids

The organic acids detected in pepper seeds were acetate, citrate, lactate, and malate, whereas formate, fumarate, and succinate were not detected. Changes in the concentrations of organic acids were observed for citrate and malate, which were significantly accumulated (by 20.04% and 17.88%, respectively) in R LED-irradiated seeds compared to the Controls (Figure 5D). 

#### 2.5.4. Other Metabolites

Interestingly, other metabolites such as chlorogenate and trionelline were not detected although they were analysed, but choline concentration was reduced by 13.19% in R LED-lighted seeds relative to Controls (Figure 5E).

### 2.6. Principal Component Analysis (PCA)

**Figure 6:**
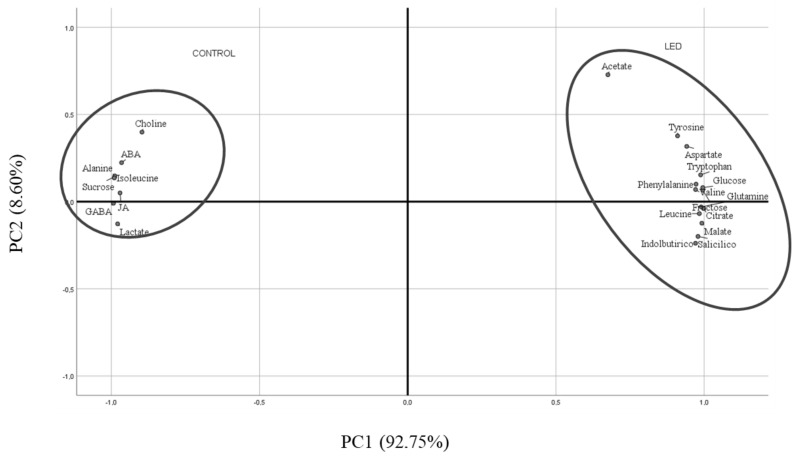
Bi-plot of PC1 viz. PC2 from principal component analysis showing the main metabolites characterising Control (CON, unlighted) and irradiated R LED (LED) pepper seeds.

For a better and simpler visual interpretation of all the data (metabolites for the different experimental repetitions of Control and R LED-irradiated seeds), a principal component analysis (PCA) was conducted (Figure 6). The PC1 component explained 92.75% of the variability observed, showing that the variability was fundamentally due to acetate, Tyr, Asp, Trp, Phe, Val, Glu, Leu, glucose, malate, citrate, SA, and IBA in R LED-irradiated seeds. PC2 (8.60%) was positively associated with Control seeds and metabolites as choline, ABA, ALA, ILE, GABA, sucrose, JA, and lactate were represented.

## 3. Discussion

The rapid and uniform seedling emergence and growth is a basic requirement for an adequate crop yield and quality. In different reports it has been shown that the time course of water uptake by seeds during germination includes three phases [2]. The results of this study clearly showed these three phases for pepper seed germination and Red LED irradiation greatly modified the kinetics of water uptake by pepper seeds, increasing not only the amount of absorbed water, but also the rate of water uptake during Phase I and Phase II of germination.

Sodium azide (NaN_3_) was reported to induce intracellular acidosis by blocking respiration via the cytochrome pathway. This acidosis results in H^+^-dependent closure of PIPs [48,49], thereby inhibiting water permeability across cell membranes [50]. In pepper seeds, NaN_3_ reduced water uptake in Control and R LED-irradiated seeds, especially during Phase III. Different studies have indicated the importance of AQPs in seed imbibition and subsequent germination [10,51,52,53]. The function of seed AQPs may be related to the water imbibition and activation of the metabolic system in the seeds, which results in higher germination [52]. However, Vander Willigen et al. [15] applied mercury as an AQP inhibitor and found that AQPs are not involved in the early imbibition phase (Phase I). Similarly, sodium azide had no significant effect on the water uptake of pepper seeds during the Phase I with regard to Control, but it reduced the water adsorption when the seeds were previously irradiated with R LED light. It was shown that R LED light increased the activity of cytochrome c oxidase (CCO), the terminal enzyme of the electron transport chains of mitochondria [54]. However, the exact mechanism by which LED irradiation enhanced photorespiration in plants is still unclear. In human cells, it has been proposed that R LED light induced the absorption of a photon by the copper subunit of the CCO [55], which enhances the ability of the mitochondria to increase the rate of oxidative phosphorylation [56]. 

In addition, Quiroga et al. [57] found that some insensitive AQPs (ZmPIP2;4, ZmPIP2;5 and ZmTIP1;1) increased their protein levels in maize plants when they were treated with NaN_3_ under normal irrigation. Therefore, the observed suppressed effect of R LED light on NaN_3_ inhibition of seed hydration from the first 10 h could be due in part to the recovery in mitochondria function, modulating pH-dependent gating of sodium-azide sensitive AQPs, together with an enhancement of sodium azide-insensitive AQP activity. 

The kinetic of water uptake was also faster under R LED light treatment, from 18 h to 48 h (plateau phase or phase II), compared with the rest of treatments. These results could be explained by previous findings where far lower concentrations of reactive oxygen species (ROS) and higher intracellular calcium ([Ca^2+^]i) were recorded in mitochondria actively making ATP [58]—such as under R LED irradiance, leading to higher AQP activity [59,60], and thereby faster water movement into seeds. Finally, during Phase III, the phase most influenced by AQP activity, faster and more effective hydration of the embryonic tissues may lead to a reduction of the germination time [61], which is the case of our R LED-irradiated seeds where GSI was significantly higher than Controls.

In this work, radicle emergence occurred from 48 h after imbibition in both irradiated and Control seeds, being the percentage higher in the former at all measured times and the exponential radicle protrusion earliest (from 60 h, compared to 67 h for Controls), reflecting a faster cell-wall loosening and greater water uptake for cell elongation. 

Cellular expansion during Phase III of germination is driven by important water uptake enabling radicle emergence through both the loosened endosperm and testa [62,63]. At 96 h of imbibition, the significantly higher humidity of R LED irradiated seeds also confirmed their higher capacity for water absorption during radicle emergence compared to Controls, without affection of seed viability. 

Several works have reported the role of different AQP isoforms in seed water uptake during radicle emergence [12,15,23]; however, this study is the first to describe effects of R LED light on AQP gene expression during that process. Interestingly, here differences in the expression pattern of all pepper AQP isoforms in response to R LED irradiance were observed inside and between treatments. Obroucheva [64] reported that *PIP* and *TIP* genes were the most strongly activated AQPs during radicle emergence, due to their implication in providing sufficient water entry into embryo-axis cells that are beginning to elongate. These results were in accordance with our results for both Control and R LED-irradiated seeds. In Control seeds, higher levels of transcripts were observed for *TIP1;7*, *TIP1;8*, *TIP3;1*, and *TIP3;2* isoforms with regard to the *NIP1;1*. *TIP* genes were functionally mainly related to the water transportation required for the enzymatic metabolism of storage nutrients in protein storage vacuoles (PSVs), process that directly preceded cell elongation [17,65], maintaining pressure potential in the vacuolar lumen (VL) at the first hours of radicle emergence [66]. However, whereas *TIP3;1* was shown to facilitate water transport [67], *TIP3;2* was found to facilitate transport of the osmolyte glycerol rather than water [68]. Control seeds showed also high expression levels of *PIP1;4*, *PIP1;5*, *PIP2;5*, and *PIP2;8* isoforms after 96 h of imbibition. Alterations in *PIP1s* and *PIP2s* gene expression may have a crucial role in governing effective water transport from cell to cell in the expanding tissue after radicle protrusion [61]. *PIP1;4* and *PIP1;5* isoforms were reported to pre-exist in dry seeds, participating in their development, with the expression being maintained at high levels in mature seeds, as reported by Shiota et al. [69]. Here, it seems that these two isoforms were also implicated in all the process of seed germination. In contrast, the expression of *PIP2;5* and *PIP2;8* isoforms were strongly increased in the embryo of rice seeds only a few hours before radicle emergence, suggesting their involvement in that process [52]. In R LED-irradiated seeds, in addition to those of Controls, three TIP (*TIP1;4*, *TIP1;6*, and *TIP4;1*) and *PIP2;3* isoforms also showed higher expression regarding *NIP1;1*. It has been shown that *TIP4;1* had dual functions in both glycerol and water transport [68], in which the transcript abundance became more important after radicle protrusion, indicating its involvement in the rapid expansion of radicle cells [13,23]. Similarly, *PIP2;3* was not expressed until radicle emergence in germinating rice seeds, followed by increased expression with seedling growth, suggesting it functions in seedling establishment rather than seed germination [52]. These findings suggest that R LED-irradiated seeds expressed some of the AQPs involved in the post-germination radicle growth. Comparing both non-irradiated and R LED-irradiated seeds, *PIP2;3* and *PIP2;5* isoforms were significantly more expressed in the latter, whereas *PIP1;5* and *PIP2;8* expression was significantly lower. These results showed a transition in the expression of AQP genes from those implicated in early seed germination phases to those involved in radicle emergence and growth. This expression pattern was in consonance with a faster germination rate in R LED-irradiated seeds. The fact that *PIP2;8* expression—which was showed to be expressed before radicle emergence—was lower in R LED-irradiated seeds, indicating the involvement of *PIP2;8* in this process. Regarding TIP isoforms, lower transcript abundance was observed for *TIP1;7*, *TIP1;8*, *TIP3;1*, and *TIP3;2* in R LED-irradiated seeds, relative to Controls. It has been reported that expression of *TIP* genes in PSVs ceased when radicles emerged from the seed coat and these proteins disappeared in parallel with the transformation of PSVs to LVs [17]; they were most likely replaced by other TIP isoforms [70]. From our results, it appeared that R LED light accelerated LV biogenesis in the embryonic axes of pepper seeds for the accumulation of endogenous osmotic solutes in elongating cells. 

Differential gene expression for *NIPs* was also observed in this work, where *NIP1;1* and *NIP4;2* were significantly lower in R LED-irradiated seeds, while *NIP4;5* was significantly higher. *NIP1;1* is localised in the plasma membrane [71], with a developmental pattern that closely resembles *TIP3;1* and *TIP3;2* [72,73]. Therefore, the involvement of *NIP1;1* with TIP3 in a developmentally controlled compensation/complementation of PIPs during early seed germination cannot be discarded. It also showed to be important for direct membrane traffic from the endoplasmic reticulum to the tonoplast [74,75]. Therefore, the lower expression of this isoform in R LED-irradiated seeds seems to be the result of reducing the delivery of proteins to the transformed PSVs after radicle emergence. Finally, our results showed a significantly higher expression of *XIP1;1* isoform in R LED-irradiated seeds, even though its function in seed germination is not yet determined. We could state that R LED-irradiated seeds are in a more advanced statement of radicle emergence with regard to Controls, as higher AQPs isoforms involved in water uptake for cell elongation rather than for stored reserve mobilisation were observed compared to Control, manifested by greater *PIP* and lower *TIP* gene expression.

In accordance with the transcript profiles of AQP isoforms, R LED light also induced metabolic changes in pepper seeds at 96 h of imbibition, in order to fulfil the growing energy demand of the developing embryo and endosperm, and to provide sufficient osmotic pressure in elongating cells. Indeed, R LED-irradiated seeds accumulated more fructose than Controls that showed higher levels of sucrose. It has been reported in germinating *Glycine max* [76], *Moringa oleifera* [77], and *Quercus ilex* [78] seeds that sucrose, the most abundant reserve carbohydrate in quiescent seeds, was conversed to fructose and glucose throughout germination and early seedling development. Therefore, the substantial rate of import of the products from reserve mobilization into glycolysis in R LED-irradiated seeds could increase the flux of pyruvate into the tricarboxylic acid (TCA) cycle [79], thereby providing more energy, as a carbon source, for faster radicle emergence [80,81]. Also, the content of total sugars was higher for Controls than for R LED-irradiated seeds, which explains their higher expression in *TIP* genes involved in the remobilization of carbohydrates. 

An advanced metabolome in R LED-lighted seeds relative to the Controls was also observed in our study by significantly higher levels in key intermediates for TCA and glyoxylate cycles, citrate, and malate, which also implies a higher energetic metabolism that would be required for better seed germination performance [82]. In contrast, the significantly lower levels of alanine and isoleucine in R LED-irradiated seeds suggests their incorporation into the gluconeogenesis pathway to provide energy supply for protruded seeds. Indeed, several analytical works based on ^14^C labelling showed that amino acids in germinating seeds contribute a large amount of carbon substrate to the respiratory system and sugar synthesis [83,84]. Glucogenic alanine and isoleucine are catabolized into TCA cycle pyruvate and succinyl CoA, respectively, which converts to oxaloacetate, the substrate for the gluconeogenic enzyme PEP carboxykinase [85]. However, the oxidative deamination of amino acids produces ammonia [86], which if not re-assimilated, induces defects in the TCA cycle [87]. To ensure ammonium re-assimilation, glutamate dehydrogenase specifically in the developing embryo axis contributes ammonium delivery to glutamine synthetase for glutamine synthesis, in the absence of primary NO_3_^-^ assimilation [86]. In turn, after being transported into the cytosol, citrate can be metabolized by aconitase to support nitrogen assimilation by producing glutamate or glutamine as an end-product [88,89]. Thus, compared to Controls, the significantly higher glutamine content in R LED seeds at 96 h of imbibition suggested greater nitrogen assimilation [90]. Citrate was also shown to mitigate drought, salinity, temperature, and heavy metal stresses in a variety of plant species [91]. Thus, when growing directly in soil, previous R LED-lighted seeds pepper will probably show better tolerance to these abiotic factors than Controls. However, this fact cannot be deduced from the present data and new studies are needed. The results of metabolic profiling showed that R LED light significantly stimulates gluconeogenesis and mitochondrial respiration in germinating pepper seeds, which provides sufficient energy needed for faster cell elongation and radicle protrusion.

The tetrazolium (2,3,5-triphenyl-tetrazolium chloride) test is used to assess seeds’ viability based on the ability of mitochondrial respiration through the electron transport chain [92]. Living cells in seed tissue use the hydrogen released by dehydrogenase enzymes during the chemical reduction of tetrazolium to form triphenyl-formazan, a red, stable, and not diffusible compound [93]. Here, no significant difference between the treatments was observed regarding seed viability, confirming that a pre-germination pulse of R LED light for pepper seeds did not affect their ability to germinate and establish their seedlings. In this study, pepper seeds showed a preferential photoblastic response, as they germinated under both irradiated and not irradiated conditions, as observed for *Murdannia nudiflora* seeds [94], being the GP significantly higher in R LED-lighted seeds at 96 h of imbibition. Similar results were obtained by Wang et al. [95], where R LED light promoted the seed germination of *Momordica charantia* (bitter gourd), and kept the germination potential at a high level. The light induction of germination is exclusively mediated by phytochrome B and other phytochromes that are maximally induced by a saturating pulse of monochromatic R light [96], in which the photoreceptor pigment is activated as a switch between 640 nm and 670 nm. The absorption of R light converts the inactive cytosol-localized P_r_ form of phytochrome (which inhibits germination) to the active nucleus-accumulated P_fr_ form (which promotes germination) [97]. Previous studies have reported that phytochrome light perception regulates the germination of dicot seeds via the modulation of expression of genes involved in phytohormone metabolism [98,99]. Here, at 96 h of imbibition, ABA was detected only in Control seeds, but not in R LED-irradiated ones. It has been reported that R light induces the expression of the gene CYP707A2 via phytochrome B, which provided ABA degradation and inactivation in both embryo and endosperm during germination [100,101]. In addition, a loss of ABA extrusion from the hypocotyl-radicle transition zone in *Medicago truncatula* seeds was observed to delay radicle emergence by preventing cell-wall loosening and cell elongation [102], which could be the case in our Control seeds. JA levels were significantly lower in R LED -lighted seeds than in Controls. JA and its derivates were found to inhibit seed germination by disrupting the peroxisomal ATP binding cassette transporter or the core β-oxidation process [103]. However, in accordance with our results, a promoting effect of R light on adventitious root initiation was also observed in *Picea abies* seedlings, likely by reducing JA biosynthesis [104]. It is important to note that JA generally works in synergy with ABA during germination through a crosstalk signalling, where ABA acts as suppressor and JA enhances ABA function [105,106]. In contrast to JA, the SA concentration was higher in R LED-lighted seeds with regard to the Controls. An increase in SA levels was also reported in *Pachyrhizus erosus* grown under R LED-light irradiation [107] and in soybean sprouts germinated under R light [108], as a result of PAL stimulation and IC synthase gene up-regulation, which are enzymatic pathways for SA biosynthesis. The accumulation of SA under R light was found to induce SA signalling, mediating the production of ROS [109]. Among ROS, hydrogen peroxide (H_2_O_2_) had a direct or indirect negative effect on ABA transport from the cotyledon to the embryonic axis, resulting in a decrease in ABA, which induced a mitogen-activated protein kinase (MAPK)-mediated reduction in the ethylene precursor 1-aminocyclopropane carboxylic acid, favouring epicotyl and radicle emergence [110]. Many reports have also described an antagonistic interaction between the SA and JA pathways [111,112]. SA was also reported to stimulate the accumulation of glycine betaine (GB), providing a nitrogen source for better germination [113], protecting the membrane functions, and increasing the activity of the antioxidant system [114]. However, GB biosynthesis occurs mainly through the degradation of choline [115], which could explain the significantly lower levels of choline in our R LED-irradiated seeds. Here, in opposition to ABA, IBA was only detected in R LED seeds. IBA is an IAA precursor which is converted into active IAA through a β-oxidation process in the peroxisome [116]. However, IBA β-oxidation releases not only free IAA, but also acetyl-CoA [117]. Thus, because IBA is important for early seedling growth, a stage in which peroxisomal activity is high in metabolizing storage oils [118], and because no amounts of IAA were detected, it may be possible that IBA is used in R LED seeds to provide energy to drive embryo growth. As unexpected, no GA3 or 6-BA was detected in both Control and R LED-lighted seeds at 96 h of imbibition, suggesting that GA3 and 6-BA are either not produced until the radicle emerges or are produced at extremely low levels, below the detection limit of our equipment. The lack of IAA could also induce a negative feedback on the biosynthesis of GA3 [119]. Taken together, R LED light adjusts phytohormone metabolism to be in consonance with the advanced germination stage of irradiated seeds.

Taking all metabolites into consideration, differences in the influence of those on Control and R LED-irradiated seeds are clear, with metabolites involved in the advanced phases of germination and early seedling development conditioning R LED-irradiated seeds.

## 4. Materials and Methods

### 4.1. Plant Material and Treatments

The seeds of sweet pepper (*Capsicum annum* L. var. Medrano F1) from Ramiro Arnedo S.L were used for all experiments. For different measurements, seeds were lined in transparent Petri dishes with moist Whatman filter paper (Merck, Darmstadt, Germany). Then, R LED light was applied for 15 min. After that, 30 mL of distilled water was added to cover the seeds for assays, except for viability determination, where the seeds were placed in a recipient with 200 mL of distilled water. 

For kinetic imbibition measurement, the initial imbibition time was counted after 15 min of water addition.

For the rest of measurements and after water addition, the seeds were place in a germination chamber (in darkness at 25 °C, 60% RH) until 96 h after starting imbibition (seeds with radicle or rootlets were analysed). Control seeds (CON) were established in similar conditions without the R LED light-pulse application. The number of repetitions of each treatment (CON and R LED) as well as the amount of the seeds in each determination was variable and depended on the analysed parameter (see distinct methods sections).

### 4.2. Red LED Light Design

**Figure 7:**
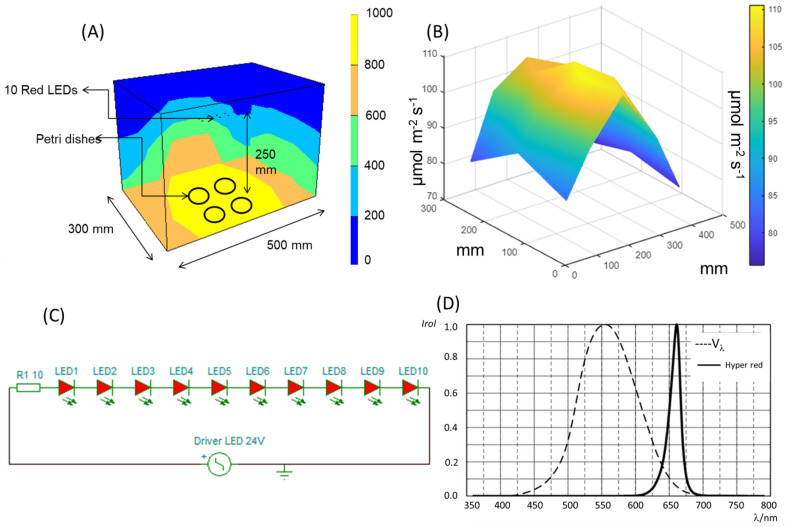
Presentation of the (**A**) dimensions, main components, operation workflow, and (**B**) spatial distribution of the photosynthetically active radiation (PAR, µmol m^−2^ s^−1^) on the modulable spectrum plant experimental chamber (MSPEC) inner surface for 10-LEDs luminaires, as well as (**C**) the 10-LED circuit diagram used in the experiment. (**D**) Osram GH CSSRM5.24 Relative Spectral Emission (Irel = f (λ); IF = 700 mA; TJ = 25 °C.

In this work, a prototype of modulable spectrum plant experimental chamber (MSPEC) was developed for testing the effects of specific R LED light (Osram, Madrid, Spain) on the first 96 h of pepper seed germination (imbibition and radicle emergence steps). To visualize the light distribution pattern along the chamber, the photosynthetic active radiation (PAR) was measured at different coordinates on the work surface of the MSPEC for 10 R LEDs (Figure 7A). The acquired data were processed with a Matlab script (Mathworks, Natick, MA, USA), from which a graphical representation of PAR distribution values was derived (Figure 7B). Ten LEDs were used for the germination tests (Figure 7C). They provided PAR values of around 100 µmol m^−2^ s^−1^ in most of the surface which allowed the arrangement of up to four Petri dishes on it. The sensors for temperature and humidity provided data ranging from 23 °C to 25 °C and from 55% to 60% RH, respectively, on all the work area. The LED chosen was manufactured by the company OSRAM. Specifically, it was selected the GH CSSRM5.24 OSLON ^®^ Square model, a compact high-power hyper red LED with proven robustness, high reliability, long lifetime, and low thermal resistance. It presents a peak wavelength at 660 nm (Figure 7D), a typical radiant flux of 1064 mW @ 700 mA and a photosynthetic photon flux of 5.82 μol/s @ 700 mA.

### 4.3. Sodium Azide Treatment

A total of 50 seeds were placed in Petri dishes with moist Whatman paper. Before measuring the kinetic of water adsorption, half of the seeds from each treatment (CON and R LED-irradiated seeds) were immersed in sodium azide (NaN_3_) at a final concentration of 7 mM [48]. The seeds were maintained under NaN_3_ immersion during the first hour of imbibition to allow the azide to penetrate through the seed coat. After that and for the rest of time-course of measurement of water imbibition, the seeds were placed in distilled water in order to avoid azide toxicity.

### 4.4. Germination Assays

The standard germination test (SGT) was carried out according to the International Seed Testing Association (ISTA) rules [120] with modifications. For that, 50 seeds of each treatment (CON and R LED) were distributed in five rows in transparent Petri dishes with Whatman paper previously moistened with distilled water. The seeds were then placed inside a germination chamber at a temperature of 25 °C and 60% relative humidity in the dark. A total of five replications (each 50 seeds) for each treatment (CON and R LED) were considered.

Counting started from the first day. Germination was calculated using Equation (1) and expressed as a percentage, where GP is the germination percentage, n_i_ the total number of germinated seeds, and N the total count seeds sampled. The final germination was considered after 96 h when the radicle was 0.50 cm.

GP (%) = n_i_/N × 100 (1)

The germination speed index (GSI) was obtained through the methodology proposed by Martínez-Solís et al. [121]. Germinated seeds were counted daily, seeds with sprouted radicles were considered, and Equation 2 was applied, where GSI is the germination speed index, Ti is the time in hours passed between the test start and the end of the interval, and Ni is the number of germinated seeds within consecutive time intervals.

GSI = ƩNi/Ti(2)

### 4.5. Viability of the Seeds

The viability analysis was determined using tetrazolium chloride, as it was described by ISTA [120]. One hundred seeds were placed in a recipient with 200 mL of distilled water, and this was put in a water bath at a temperature of 35 °C for 14 h. Later, a 1% tetrazolium chloride solution (2 mL) was added, and the recipient with the seeds was put in a water bath at a temperature of 35 °C for 4 h. Finally, the seeds were rinsed with distilled water and examined under a microscope (LEICA, EZR^®^, L’Hospitalet de LLobregat, Spain). The embryos were classified according to colour intensity: (1) alive with high vigour, when they were completely dyed with an intense red colour, (2) alive with low vigour, when their coloration was a pale red, and (3) not viable, when they remained colourless. The classified seeds were expressed as the percentage of viable and unviable embryos. A total of five replications for each treatment were realized.

### 4.6. Imbibition Kinetics

Using an analytical balance (Model: 161 RADWAG PS 4500/C2), 25 g of seeds were weighed for each treatment and grouped on transparent Petri dishes with filter paper in 5 pools per treatment (CON, LED, azide, and LED+azide). Next, 6 mL of distilled water was added in order to moisten the filter paper. Then, R LED light was applied for 15 min. After that, 30 mL of distilled water was added to the seeds to cover them for 96 h at room temperature (23 °C). The increase in weight was registered during this time and the amount of water adsorbed (g g^−1^ DW) was expressed through Equation (3), where Wa is the water adsorbed, Wi is the initial weight, Wf is the final weight, and Hf is the percentage humidity content (that it was similar for Control and R LED-treated seeds before the start of imbibition) [122]. A total of five replications for each treatment was realized.

Wa = [(Wf − Wi)/Wf] × [1 − (Hf/100)](3)

### 4.7. RNA Extraction and Reverse Transcription

After 96 h of imbibition, 0.5 g of seeds with radicle or rootlets, per treatment and experimental replicate (*n* = 3), were frozen and ground to a fine powder in liquid nitrogen. Total RNA was extracted using the RNeasy Plant Mini Kit (Qiagen, Hilden, Germany), according to the manufacturer’s instructions. Contaminating DNA in the samples was removed with DNase I, using the DNA-free Kit (Ambion, Applied Biosystems, Austin, TX, USA), and the RNA concentration was quantified with a Nanodrop 1000 spectrophotometer (Thermo Fisher Scientific, Waltham, MA, USA). After that, the extracted RNA was stored at −80 °C until use.

The cDNA was synthesised from 2 μg of total RNA, using the M-MLV reverse transcriptase from the RETROscript Kit (Ambion, Applied Biosystems, Austin, TX, USA). Reverse transcription was carried out with heat denaturation of the RNA, according to the manufacturer’s instructions.

### 4.8. Quantitative Real-Time PCR (qRT-PCR) Analyses

To compare the expression of distinct AQP isoforms under the different treatments, qRT-PCR was performed as described by Muries et al. [123], in an Applied Biosystems 7500 Real-Time PCR system. The primers and the lengths of the amplicons for *PIP*, *TIP*, *NIP*, *SIP*, and *XIP* isoforms were those described by Uppuluri et al. [124] (Supplemental Table S1).

After denaturation at 95 °C for 10 min, amplification occurred in a two-step procedure: 15 s of denaturation at 95 °C and 1 min of annealing and extension at 60 °C, followed by a dissociation stage. Data collection was carried out at the end of each round in step 2. These conditions were used for both target and reference genes and the absence of primer dimers was checked in controls lacking templates. The amplifications were performed on three independent samples for each treatment and seeds (biological replicates) and triplicate reactions were carried out for each sample (technical replicates) in 96-well plates. The transcript levels were calculated using the 2^−ΔΔCt^ method [125], for both target and reference genes. Standard curves (log of the cDNA dilution vs. Ct) using serial 10-fold dilutions of the cDNA were built for each pair of selected primers, obtaining 95% PCR efficiency, corresponding to a slope of −3.561. Actin and ubiquitin were used as house-keeping genes for the standardisation of each sample [126]. ΔCt (target gene) calculates as Ct (target gene) − Ct (house-keeping gene). ΔΔCt was then calculates as ΔCt (target gene)—ΔCt (reference sample gene). The final result of this method is presented as the fold change of target gene expression in a target sample, relative to a reference sample (CON *NIP1;1*), normalized to each reference gene. With both standard genes, similar relative expressions were obtained.

### 4.9. Metabolite Analysis by 1H-NMR Spectroscopy

A “non-targeted” metabolic analysis was conducted in the seeds with radicle or rootlets after 96 h of water hydration. For that, 0.5 g of rootlets were ground with liquid nitrogen with a mortar and pestle and lyophilized. Afterwards, the samples were prepared for analysis according to the protocol by Van de Weijer and Schrauwen-Hinderling [127]. For this analysis, a Nuclear Magnetic Resonance (NMR) system coupled to a 500 MHz Bruker spectrometer (Bruker Biospin, Rheinstetten, Germany) equipped with a broadband 5 mm N_2_ CryoProbe Prodigy BBO. The seed extracts were measured at 300.1 ± 0.1 K without rotation and with 4 test scans before the 32 scans performed for the experiment.

The acquisition parameters were set in the following manner: the size of the FID = 64 K, spectral band = 12.4345 ppm, receiver gain = 28.5, acquisition time = 2.18 s, relaxation delay = 2 s, and line broadening = 0.50 Hz. The acquisition of data was performed through the NOESY pulse sequence of pre-saturation (Bruker 1D, noesypr1d) with water suppression through the irradiation of the water frequency during the recycling and mixing times. In the processing of the samples and for each spectrum separately, a reduction of noise was produced, based on the deconvolution of the multi-level signal. Afterwards, a correction was performed of the baseline, and to complete the process, an interpolation technique of the areas of the signal was utilized.

All of this provides us with a “fingerprint” of the sample, a general view of the metabolites that are most represented produced by the cells at time of harvest, expressing the chemical shifts (d) in parts per million (ppm). The NMR equipment detects the signals and records them as frequency versus intensity graphic, known as the “acquisition spectrum”.

The resulting 1H-NMR spectra were processed with the Chenomx NMR Suite program version 8.3 (Chenomx, Edmonton, AB, Canada), in order to identify and quantify the metabolites of interest. All the samples were calibrated with the signal from the internal standard (IS), the deuterated Trimethylsilylpropionic acid sodium salt (TSP-d4) and the pH was set to a value of around 6. The software utilized includes a broad range of spectrum data that can be utilized to detect the metabolites that are over 5–10 mM: among the metabolites that were found and/or quantified, the following are highlighted: Alanine, Aspartate, Asparagine, Glutamate, Glutamine, Isoleucine, Leucine, Phenylalanine, Proline, Tryptophan, Tyrosine, Valine, Acetate, Citrate, Lactate, Malate, Fructose, Glucose, Sucrose, GABA, and Choline. A total of five replications for each treatment were realized.

### 4.10. Hormone Analysis by UPLC-QToF-MS/MS

#### 4.10.1. Sample Preparation

Sample preparation was carried out according to Müller and Munné-Bosch [128]. For that, frozen material (about 100 mg fresh weight of seeds with radicles or rootlets) was ground in liquid nitrogen with the mixer mill MM400 (Retsch GmbH, Haan, Germany) in a 2 mL Eppendorf tube, and then extracted with 1 mL of extraction solvent (methanol:isopropanol, 20:80 (*v*/*v*) with 1% of glacial acetic acid) using ultra sonication (4–7 °C). The labelled forms of the compounds d4-SA, d6-ABA, d5-JA, d5-IAA, d2-GA1, d2-GA4, d2-GA9, d2-GA19, d2-GA20, d2-GA24, d4-ACC, d6-2iP, d6-IPA, d5-Z, and d5-ZR were added as internal standards. D5-Z and d5-ZR were used as internal standards for DHZ and DHZR, respectively. After centrifugation (10,000 rpm for 15 min at 4 °C), the supernatant was collected, and the pellet was re-extracted with 0.5 mL of extraction solvent, and the extraction repeated three times again. Then, supernatants were combined and dried completely under a nitrogen stream and re-dissolved in 300 μL of methanol, centrifuged (10,000 rpm for 5 min) and filtered through a 0.22 μm PTFE filter (Waters, Milford, MA, USA). Samples (5 μL) were then analysed by UPLC/ESI-MS/MS. Hormones were determined in five independent samples for each treatment. Quantification was done by the creation of calibration curves including each of the 17 unlabelled analyte compounds (SA, ABA, JA, IAA, GA1, GA4, GA9, GA19, GA20, GA24, ACC, 2iP, IPA, Z, ZR, DHZ, and DHZR). Ten standard solutions were prepared ranging from 0.05 ng mL^−1^ to 1000 ng mL^−1^ and for each solution a constant amount of internal standard (as described above) was added. Calibration curves for each analyte were generated using Analyst™ software (Applied Biosystems, Inc., California, USA). The limit of detection (LOD, S/N = 3) and the limit of quantification (LOQ, S/N = 10) were also calculated with the aid of this software. 

#### 4.10.2. UPLC-QToF-MS/MS Analysis

UPLC-QToF-MS/MS was performed using a Waters ACQUITY UPLC I-Class System (Waters Corporation, Milford, MA, USA) coupled to a Bruker Daltonics QToF-MS mass spectrometer [maXis impact Series with a resolution ≥ 55,000 FWHM *Bruker Daltonics*, Bremen, Germany], using ESI for both positive- [ESI (+)] and negative- [ESI (−)] ionization modes. UPLC separation was performed using an HSS T3 C18 100  ×  2.1 mm column with 1.7 µm of size particle (Waters Corporation, Milford, MA, USA) at a flow rate of 0.4 mL min^−1^. The separation was performed using H_2_O with acetic acid (AcOH) at 0.05% (pH ~3.20) (PanReac AppliChem, Barcelona, Spain) as the weak mobile phase (A) and ACN with AcOH at 0.05% (J. T. Baker, Morristown, NJ, USA) as the strong mobile phase (B). The gradient started with 1% of B at 0 min, which was progressively increased to 99% at minute 5; after that, it was held until minute 7.50 and then decreased to 1% at 10 s, where it remained until minute 10.50. Nitrogen was used as the desolvation gas with a flux of 9 L min^−1^ and as the nebulizing gas with a pressure of 2.0 bars. The drying temperature was 200 °C and the column temperature was 25 °C. The voltage source was 4.0 kV for ESI (−) and 4.5 kV for ESI (+). The MS experiment was carried out using HR-QTOF-MS, applying 24 eV for ESI (+) and 20 eV for ESI (−) and using broadband collision-induced dissociation (bbCID). MS data were acquired over an *m/z* range of 50–1200 Da.

The external calibrant solution was delivered by a KNAUER Smartline Pump 100 with a pressure sensor (KNAUER, Berlin, Germany). The instrument was calibrated externally before each sequence with a 10 mM sodium formate solution. The mixture was prepared by adding 0.5 mL of formic acid and 1.0 mL of 1.0 M sodium hydroxide to an isopropanol/Milli Q water solution (1:1, *v*/*v*). A total of five replications for each treatment were realized, considering each replication a different sample extraction.

### 4.11. Data Analysis

The statistical analysis included a one-way ANOVA with the SPSS program v24. When the ANOVA was significant (*p* < 0.05), Tukey’s HSD test for the separation of means was applied for *p* < 0.05.

A principal component analysis (PCA) was carried out to identify and classify the principal metabolites to be used in distinguishing Control and R LED-irradiated seeds. This PCA was conducted on the mean of distinct values recorded for different metabolites, from five tests for each treatment (Control and R LED) using SPSS (PASW Statistics18.0) computer package (SPSS 2006).

## 5. Conclusions

R LED light applied to pepper seeds in a short pulse enhanced the kinetics of water uptake by seeds at all germination phases, increasing their germination performance. The symplastic water movement for embryo growth during germination was modulated by the R LED irradiance. Indeed, a faster vacuolization manifested by reduction in the expression of TIP genes (*TIP1;7*, *TIP1;8*, *TIP3;1*, and *TIP3;2*), together with increased water absorption for cell elongation through the higher expression of PIP genes involved in water transport (*PIP2;3* and *PIP2;5*) led to faster radicle emergence and greater GP in R LED irradiated seeds, compared to Controls. Reduced *NIP1;1* and *NIP4;2* expression could be related with the reduction of delivery of proteins from the protein storage vacuoles after radicle emergence. In any case, the role of NIP and XIP in seed germination needs further studies. The faster water uptake in R LED-irradiated seeds was in consonance with a more advanced energetic metabolism involving monosaccharides, organic acids, and amino acids, in order to satisfy the energy required for faster germination. The R LED light also affected the accumulation of the hormones of germination activating those involved in radicle emergence and growth. Therefore, after R LED irradiation, a direct link between the rates of water transport, seed carbohydrate metabolism, hormone regulation, and radicle growth can be established. If advanced germination induced by R LED light is an advantage for the plant to cope with stress in the environment, it must be studied as well as the effect of different spectral lights on seed germination stages.

## Figures and Tables

**Table 1:** Germination percentage (GP), germination speed index (GSI), percentage of seed viability, and percentage of seed humidity in Control (CON, unlighted) and irradiated R LED (LED) pepper seeds, after radicle emergence and growth during germination (after 96 h of imbibition). Values are means ± SE (*n* = 5). The different letters indicate significant differences among treatments according to Tukey’s test (*p* < 0.05).

	GP (%)	GSI	Viability (%)	Humidity (%)
CON	88 ± 1.63 b	0.35 ± 0.03 b	86 ± 4 a	2.64 ± 0.18 b
LED	93 ± 1.91 a	0.37 ± 0.03 a	90 ± 5 a	3.27 ± 0.20 a

**Table 2:** Salicylic acid (SA), indole-3-butyric acid (IBA), indole-3-acetic acid (IAA), gibberellic acid (GA3), 6-Benzyladenine (6-BA), abscisic acid (ABA), and jasmonic acid (JA) concentrations (ng mL^−1^) in Control (CON, unlighted) and R LED (LED)-irradiated pepper seeds after 96 h of imbibition. Values are means ± SE (*n* = 5). The different letters indicate significant differences among treatments according to Tukey’s test (*p* < 0.05). n.d. Not detected.

	SA	IBA	IAA	GA3	6-BA	ABA	JA
CON	88.04 ± 1.59 b	n.d	n.d	n.d	n.d	13.44 ± 1.13	648.67 ± 6.35 a
LED	116.24 ± 2.58 a	7.26 ± 0.23	n.d	n.d	n.d	n.d	573.11 ± 10.95 b

## Data Availability

Not applicable.

## Supplementary Materials

The following supporting information can be downloaded at: https://www.mdpi.com/article/10.3390/ijms24054779/s1.
