# Supplementary material for: Red LED Light Improves Pepper (Capsicum annuum L.) Seed Radicle Emergence and Growth through the Modulation of Aquaporins, Hormone Homeostasis, and Metabolite Remobilization
Source: Int J Mol Sci. 2023 Mar 1;24(5):4779. doi: 10.3390/ijms24054779 (PMC10002511; doi:10.3390/ijms24054779)
Supplement: Supplementary file 1 [file ijms-24-04779-s001.zip › ijms-2237204-supplementary.pdf]

Supplemental Table S1. List of CaAQP gene primers used for gene expression

**CaAQP isoforms**	**Forward Primer**	**Reverse Primer**
CaNIP1;1	TCAGTAGTGGTGAATGCAGATAAA	GCAAGTGGCAAAGGCAATAG
CaNIP1;2	TCTATGGGTTTACTTGTTAGGC	GGCCTAACAAGTAAACCCATAGA
CaNIP3;1	CGTTGTCACACTACCTGGAAT	CAATGGTAACAGCAGGGTTAAAG
CaNIP4;1	CACGTATCGGGTGGTCATTT	GACCCTGTTACCTGAGCTATTATG
CaNIP4;2	CAGGGATATGTGTGACATGGG	GACGGAAGAGGGTGAAAGTAATAG
CaNIP4;5	GCCAGTATCAGGAGCATCAA	GCCCAACTATGTAAACCCAAAG
CaNIP4;6	GGAGAACTCACTGGACTTGTTAT	CAAACTTCTTGCGGGATTCATT
CaNIP6;1	GACCGTCATGCTCAACATACT	CTTGTAGTTTCCTGCTGCTACT
CaNIP7;1	GAACCCAGCCAGGTCTTTAG	CTCCTGCAACAGCTCCTATAAT
CaPIP1;1	GGCCTGGACGCTGAAATAA	CAAGACTCCTAACTGGGTTGATAC
CaPIP1;2	GGATTTGCGGTGTTCCTAGT	TGCGGGTTCCTTGTTATAGATG
CaPIP1;3	AGAGACTCCCATGTCCCTATT	TAATGCCAGTGCCTGTGATAG
CaPIP1;4	CGGTGTTCTTGGTTCATTTGG	AGCTTGGTCTTGGTTGAAGAT
CaPIP1;5	GTATCTCAGGAGGACACATCAAC	CCAAGGCACTGCATCACTAT
CaPIP2;2	ATAGCACGGGTACTGGTTTG	GAGTCTCTGGCGTTTCTCTTAG
CaPIP2;3	TGGCCACCATCCCAATTAC	CCATTGGTCCAACCCAGAATA
CaPIP2;4	TGTGGTGGTGTTGGTATTCTT	GGGTTAATGTGTCCTCCAGAAA
CaPIP2;5	GATGGTGTTGGTATTCTTGGTATTG	TGTCACTGCTGGGTTAATATGT
CaPIP2;6	ATAGCACGGGTACTGGTTTG	GAGTCTCTGGCGTTTCTCTTAG
CaPIP2;7	TGGTTCACTTAGCCACCATAC	CGCCTTGTCACCATTGTAAATAA
CaPIP2;8	GTCTTGGCACCACTTCCTATT	AAACTACGAGCCGGGTTAATAC
CaTIP1;2	CTTGGGTGGTGGACTTCTTT	CGAATGGACCGAGCCAATAA
CaTIP1;4	AGCAATGAACCCTGCTATGG	AATGGTAGCACCCACGAATG
CaTIP1;6	CCTTTCTGGGAGGTCACATTT	GCAATCTTGAGGAGGAAGAGAG
CaTIP1;7	AGCAATGAACCCTGCTATGG	CGAATGGACCGAACCAGTAAA
CaTIP1;8	CCTTTCTGGGAGGTCACATTT	GCAATCTTGAGGAGGAAGAGAG
CaTIP1;10	CCAAAGAGACAACCGCTATGA	GTTCACATGACCTCCCGATATG
CaTIP1;11	GGCTGATTTGGGAGGCAATA	CCAAATGTGACAGCAGGATTTAC
CaTIP1;12	GCGGTTGGTGCTAACATTTC	AAGCAATTGAGCAACCCAATAC
CaTIP1;13	TGCTTGCTCCTCAAGTATTCC	GCCCAAATGTCGTCACTATCT
CaTIP2;1	TTAACCCTGCTGTCACCTTC	GAGGAGGATGCAAGCTACAA
CaTIP2;2	GCTCTCTTTGTGGCTGTTTCT	GTCCGGTAAGGATGGTGATTTG
CaTIP2;3	TGAACCCAGCTCGATCATTT	CCATAAATAAACCCGGCCAATC
CaTIP3;1	GTCCTTGTAGGTGGTCCTTTC	TCCTCCATCTCCAGCCTATAA
CaTIP3;2	GCGCATGCTTTATCGCTATTT	AGGTAACAGCTGGGTTGATG
CaTIP4;1	GAGATCCACTGGTGAGCTTATT	AGTGTAACAGCAGGGTTGAG
CaTIP5;1	GGGATCTATGAACCCTGCTTAC	TGCAGGGAAGACCACATTATC
CaSIP1;1	CCTCTTCATCACCACCCTTATT	TCCAAGACCAGCAGCATAAA
CaXIP1;1	GGCCATGTCACTGTATTGTCTA	GAAACACCTAGCCGGATTCA
CaXIP1;2	GGCAGGCATTCTTGTGTTTATT	AGCACCTAGCTGGATTCATTC
CaUBI-3	TGTCCATCTGCTCTCTGTTG	CACCCCAAGCACAATAAGAC
Ca ACT	TGTTATGGTAGGGATGGGTC	TTCTCTCTATTTGCCTTGGG
